# Structure of Main Protease from Human Coronavirus NL63: Insights for Wide Spectrum Anti-Coronavirus Drug Design

**DOI:** 10.1038/srep22677

**Published:** 2016-03-07

**Authors:** Fenghua Wang, Cheng Chen, Wenjie Tan, Kailin Yang, Haitao Yang

**Affiliations:** 1 School of Life Sciences, Tianjin University, Tianjin 300072, China; 2 Tianjin International Joint Academy of Biotechnology and Medicine, Tianjin 300457, China; 3 Key Laboratory of Medical Virology, Ministry of Health, National Institute for Viral Disease Control and Prevention, Chinese Center for Disease Control and Prevention, Beijing 102206, China; 4 Cleveland Clinic Lerner College of Medicine of Case Western Reserve University, Cleveland, OH 44195, USA

## Abstract

First identified in The Netherlands in 2004, human coronavirus NL63 (HCoV-NL63) was found to cause worldwide infections. Patients infected by HCoV-NL63 are typically young children with upper and lower respiratory tract infection, presenting with symptoms including croup, bronchiolitis, and pneumonia. Unfortunately, there are currently no effective antiviral therapy to contain HCoV-NL63 infection. CoV genomes encode an integral viral component, main protease (M^pro^), which is essential for viral replication through proteolytic processing of RNA replicase machinery. Due to the sequence and structural conservation among all CoVs, M^pro^ has been recognized as an attractive molecular target for rational anti-CoV drug design. Here we present the crystal structure of HCoV-NL63 M^pro^ in complex with a Michael acceptor inhibitor N3. Structural analysis, consistent with biochemical inhibition results, reveals the molecular mechanism of enzyme inhibition at the highly conservative substrate-recognition pocket. We show such molecular target remains unchanged across 30 clinical isolates of HCoV-NL63 strains. Through comparative study with M^pro^s from other human CoVs (including the deadly SARS-CoV and MERS-CoV) and their related zoonotic CoVs, our structure of HCoV-NL63 M^pro^ provides critical insight into rational development of wide spectrum antiviral therapeutics to treat infections caused by human CoVs.

Coronaviruses (CoVs) are a diverse group of enveloped positive-strand RNA viruses in the family *Coronaviridae*^1^,^2^. CoVs have been identified in a wide variety of hosts, including mammals and birds, and are shown to cause a number of respiratory and enteric diseases^1^,^3^,^4^. In 2003, the global epidemic of an atypical form of pneumonia named severe acute respiratory syndrome (SARS) led to the discovery of SARS-CoV, a previously unknown CoV, as the etiologic pathogen^5^,^6^,^7^,^8^. Started in South China, SARS outbreak quickly resulted in more than 800 deaths worldwide^9^. Patients with SARS-CoV infection developed diffuse alveolar damage with the potential to progress into acute respiratory distress syndrome and eventually death^10^. Almost 10 years later, another previously unknown CoV, Middle East respiratory syndrome coronavirus (MERS-CoV), was found to cause a new epidemic starting in the Arabian Peninsula in 2012^11^,^12^,^13^. MERS infection led to acute pneumonia and renal failure, with mortality rate as high as 50% in hospitalized patients^14^,^15^. In addition to the deadly SARS-CoV and MERS-CoV, 4 other human CoVs have been identified so far, namely HCoV-229E, HCoV-OC43, HCoV-NL63, and HCoV-HKU1, which are known to cause comparatively mild common colds^9^,^16^,^17^. According to their genomic sequences, these 6 HCoVs are further classified into *alphacoronavirus* genus (HCoV-229E and HCoV-NL63) and *betacoronavirus* genus (HCoV-OC43, HCoV-HKU1, SARS-CoV, and MERS-CoV)^12^,^18^. The emergence of CoV infection in human beings are believed to begin with zoonotic transmission from animal reservoirs^9^. For example, high degree of genomic sequence similarity was shown between bovine CoV and HCoV-OC43, suggesting a relatively recent animal-to-human transmission^11^,^19^. In the case of human SARS-CoV, recent studies identified several SARS-like bat CoVs with over 95% genomic sequence identity, suggesting bats as the potential zoonotic reservoir^20^,^21^,^22^. While dromedary camels are suspected to be either reservoir or vector for MERS, as genomic sequence of isolated dromedary MERS-CoV was found identical to that of human MERS-CoV^23^,^24^,^25^.

Human coronavirus NL63 (HCoV-NL63) was first isolated in 2004 from a 7-month-old child suffering from bronchiolitis and conjunctivitis in the Netherlands^16^. HCoV-NL63 has been documented to circulate in human population worldwide^26^,^27^,^28^,^29^,^30^,^31^,^32^,^33^, and is considered the causative pathogen for up to 10% of all respiratory illnesses^34^,^35^,^36^,^37^. Infected patients are typically young children with upper and lower respiratory tract infection, presenting with symptoms including croup, bronchiolitis, and pneumonia^38^,^39^. Nevertheless, infections in adults have also been reported, though consequences could be more severe in those with compromised immune system or other comorbidities^40^,^41^,^42^. Similar to SARS-CoV, HCoV-NL63 also uses angiotensin-converting enzyme 2 (ACE2) as the receptor for cellular entry^43^. Full genome sequences of HCoV-NL63 have been determined, revealing a mosaic structure with multiple recombination sites which indicate that possible mutation and recombination could occur when co-infected with other CoVs^44^,^45^. Based on molecular clock analysis, HCoV-NL63 shares common ancestry with bat *alphacoronavirus* sequences, with probable divergence 563–822 years ago^46^. However, the direct bat ancestor of HCoV-NL63 has not been found yet^47^.

Currently there are no approved antiviral drugs or vaccines against human CoV infection, though several compounds have been investigated in pre-clinical studies^9^. From a public health perspective, no effective antiviral strategy is available in face of future CoV emergence, potentially transmitted from the vast and mutable zoonotic reservoir. Previously, we have demonstrated that main protease (M^pro^) is a conserved drug target throughout the subfamily *Coronavirinae*, which is suitable for designing wide-spectrum inhibitors^48^,^49^. The 5′ two-thirds of coronaviral genome is consisted of open reading frame 1 (ORF1), which encodes two large polypeptides of the replicase machinery: pp1a, and through ribosomal frameshift, pp1ab^18^. These two polypeptides are cotranslationally cleaved into mature nonstructural proteins (Nsps) through two proteases encoded in the 5′ region of ORF1: papain-like protease (PLP) and 3C-like protease (3CL or Nsp5)^50^,^51^. 3CL protease is more commonly known as M^pro^ because of its dominant role in the posttranslational processing of the replicase polyprotein. The M^pro^s from different human and animal CoVs are known to share significant homology in both primary amino acid sequence and 3D architecture, providing a strong structural basis for designing wide-spectrum anti-CoV inhibitors^48^,^49^,^52^,^53^,^54^,^55^. They employ a similar substrate-binding pocket, usually with a requirement for glutamine at P1 position and a preference for leucine/methionine at P2 position. Interestingly, in contrast to other HCoVs, only HCoV-NL63 and HCoV-HKU1 exhibit a unique substrate preference of histidine in P1 position at the cleavage site between nsp13 and nsp14^37^,^53^. The structural and pharmaceutical significance of the P1 position preference of HCoV-NL63 M^pro^ remains to be addressed.

Here, we report the crystal structure of HCoV-NL63 M^pro^ in complex with a synthetic peptidomimetic inhibitor, N3. Structural analysis reveals relative conservation at the P1 pocket. Through comparison with M^pro^s from other CoVs, we provide structural insight into rational drug design at a conserved target across pathological human coronaviruses and their related zoonotic counterparts.

## Results

### Structural Overview

**Figure 1:**
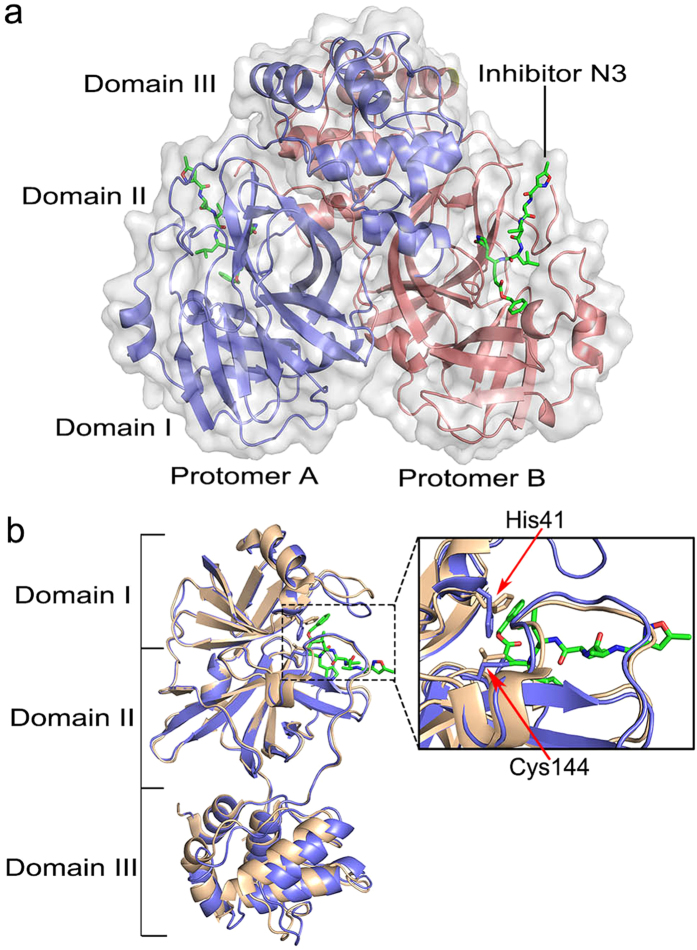
Structural overview of HCoV-NL63 M^pro^. (**a**) Overview of homodimer in one asymmetric unit (A: slate and B: deep salmon). Protomers are shown in cartoons, and N3 inhibitors are shown as green sticks. (**b**) Structural alignment of protomer A in M^pro^-N3 (slate) complex with that in apo enzyme (light orange, PDB ID: 3TLO; C. P. Chuck & K. B. Wong, unpublished work). The backbone is shown in cartoons, and N3 inhibitor is presented as green sticks.

There are two protein molecules in an asymmetric unit. The two molecules form a typical homodimer (Fig. 1a), which has been observed in the crystal structures of other CoV M^pro^s^48^,^52^,^53^. Previous studies have demonstrated the existence of M^pro^ homodimer in solution which is also the only active form of the enzyme^56^,^57^,^58^,^59^, supporting the physiological relevance of structural findings. A structural comparison of protomer A in M^pro^-N3 complex with that in apo enzyme (PDB ID: 3TLO; C. P. Chuck & K. B. Wong, unpublished work) revealed an overall architecture of three domains (Fig. 1b) in each protomer, a common feature among CoV M^pro^ structures. Domain I (residues 8–100) and domain II (residues 101–183) together form a chymotrypsin-like fold, and the substrate-binding site is located in a cleft formed between domain I and domain II. The catalytic dyad composed of Cys144 and His41 lies in the center of substrate-binding site. Domain III (residues 200–303) of HCoV-NL63 M^pro^ is composed of a globular antiparallel α-helical cluster, a unique feature of CoV M^pro^ that is required for homodimer formation. Domain III is connected to domain II through a long loop region of 16 residues. X-ray data-processing and refinement statistics are included in Table 1.

### Michael acceptor and inhibitor binding at active site

**Figure 2:**
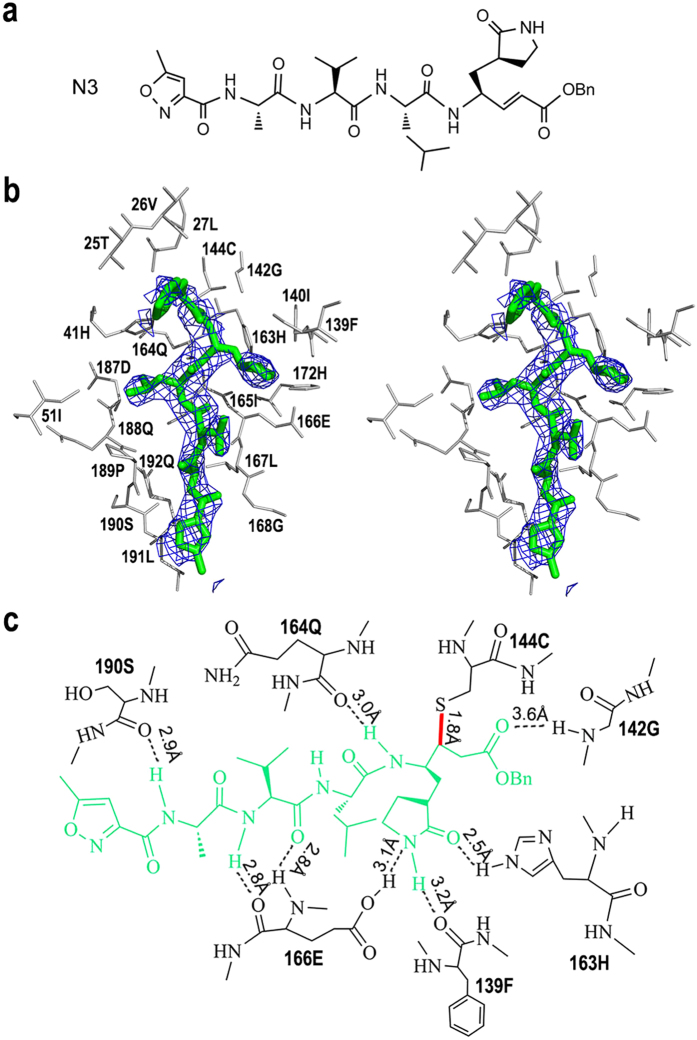
Structure of inhibitor N3 and its interaction with HCoV-NL63 M^pro^. (**a)** The structure of compound N3. (**b**) A stereo view of N3 bound to the substrate-binding pocket of HCoV-NL63 M^pro^ at 2.85 Å. N3 inhibitor is shown in green and covered by an omit map contoured at 1.0 σ. Residues forming the substrate-binding pocket are shown in silver. **(c)** Detailed view of the interaction between N3 and HCoV-NL63 M^pro^. The N3 inhibitor is shown in green. Hydrogen bonds are shown as dashed lines labeled with interaction distances. The covalent bond between N3 and Sγ of Cys144 is labeled in red.

Michael acceptor inhibitors such as N3 (Fig. 2a) undergoes mechanism-based inhibition to achieve covalent irreversible inactivation, as shown in the equation (1):


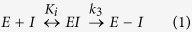


The inhibitor first forms a reversible complex (EI) with the enzyme under the equilibrium-binding constant *K*_*i*_. It then undergoes nucleophilic attack by the active site Cys of the enzyme, leading to the formation of a stable covalent bond (E-I). This step is governed by the inactivation rate constant, *k*_*3*_. Using a CoV consensus substrate reported previously^48^,^49^,^53^, we first determined the values for *K*_*m*_ and *k*_*cat*_ of the apo enzyme of HCoV-NL63 M^pro^, as 50.8 ± 3.4 μM and 0.098 ± 0.004 s^−1^, respectively (Table 2). Cross comparison with M^pro^s from other human CoVs revealed that the kinetic parameters of HCoV-NL63 M^pro^ is relatively close to those for SARS-CoV (*K*_*m*_ = 129 ± 7 μM, *k*_*cat*_ = 0.14 ± 0.01 s^−1^)^49^. Rather *K*_*m*_ of HCoV-NL63 is higher than that for HCoV-229E, while its *k*_*cat*_ is approximately ten fold larger than those for HCoV-229E and HCoV-HKU1. We then added inhibitor N3 to the kinetic assay of HCoV-NL63 M^pro^, and calculated the *K*_*i*_ and *k*_*3*_ as 11.3 ± 1.0 μM and 42.4 ± 5.0 (10^−3^∙s^−1^), respectively (Table 2). Although the *ki* for HCoV-NL63 is higher compared with those for SARS-CoV and HCoV-229E, indicating a lower affinity of inhibitor N3 to the apo enzyme, its *k*_*3*_ is significantly larger, which strongly supports the ability of N3 to achieve mechanism-based irreversible inhibition against HCoV-NL63 M^pro^.

Analysis of the complex structure of HCoV-NL63 M^pro^ bound to inhibitor N3 provides further insight into the inhibition mechanism (Fig. 2b). Since N3 binds to Protomer A and B similarly, we will only look into the binding mode of N3 in Protomer A below. Cβ of vinyl group on inhibitor N3 forms a standard 1.8 Å covalent bond with the Sγ atom of Cys144, as evidenced by clear electron density (Fig. 2b). This indicates that a Michael addition reaction occurred between N3 and the catalytic site Cys144. Compared with the apo enzyme, only one specific conformation is observed due to the formation of covalent bond while there exists a double conformation for Sγ in the apo enzyme. The carbonyl oxygen of ester on N3 is very close to the backbone NH of Gly142 and the backbone of NH of Cys144 (Fig. 2c), which mimics the tetrahedral oxyanion intermediate state formed during serine protease cleavage and provides additional support to anchor the Michael acceptor. The benzyl ester part of N3 further extends into the S1’ pocket, forming Van der Waals interaction with Val26 and Leu27 (Fig. 2b).

**Figure 3:**
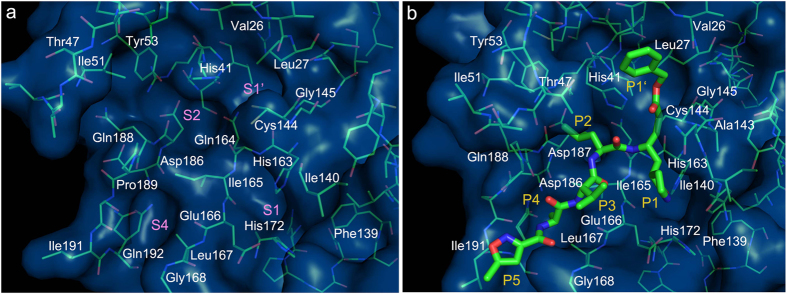
Surface representation of native HCoV-NL63 and its complex with inhibitor N3. (**a)** Surface representation of substrate-binding pockets from apo enzyme of HCoV-NL63 M^pro^ (marine, PDB ID: 3TLO). The S1, S2, S4, and S1’ pockets and residues forming the substrate-binding pocket are labeled. **(b)** Surface representation of HCoV-NL63 M^pro^ (marine) in complex with N3 inhibitor (green). Water molecules are shown as red spheres. The P1-P5, and P1’ groups of N3 inhibitor are labeled together with residues forming the substrate-binding pocket.

Comparison between the molecular surfaces of HCoV-NL63 M^pro^ complexed with N3 and the apo enzyme reveals that the pocket accommodating N3 undergoes significant conformation changes upon inhibitor binding (Fig. 3a,b) as following: (1) the imidazole group of His41 swifts ~5 Å towards the hydrophobic core of the protein to better accommodate P1’ site; (2) the main chain of residues 138–142 which constitute the outer wall of S1 subsite moves toward the lactam ring, causing the shrinking of S1 pocket; (3) residues 45–51 flip over to act as a lid to cover P2 subsite; (4) residues 164–168 and 187–191 extend into opposite directions to host N3. In the following sections, we will describe the detailed structural features of the subpockets.

### Smaller S1 pocket to accommodate P1 histidine

Coronaviral M^pro^s are known to have strong preference to glutamine (Q) at P1 site of substrate. Genome sequence analysis of HCoV-NL63 revealed that 10 out of 11 M^pro^ cleavage sites bear glutamine at P1 position, except that the recognition site between nsp13 (helicase) and nsp14 (exonuclease) where histidine replaces glutamine at P1 site^37^. This feature is unique only in genomes of HCoV-NL63 (NC_005831) and HCoV-HKU1 (NC_006577). In contrast, glutamine is the exclusive residue in P1 among all cleavage sites for the other four human CoVs, including HCoV-229E (NC_002645), HCoV-OC43 (NC_005147), SARS-CoV (NC_004718), and MERS-CoV (JX869059). Such novel substrate specificity at P1 in HCoV-NL63 and HCoV-HKU1 implies unique structural feature of S1 subpocket. The crystal structure of HCoV-NL63 M^pro^ shows that the size of its S1 pocket is comparable to that of HCoV-HKU1, but smaller than those of HCoV-229E and SARS-CoV. Clearly, a smaller S1 pocket in HCoV-NL63 could better accommodate the smaller side chain of histidine residue at P1 of nsp13-nsp14, and facilitate cleavage when weakened oxyanion hole is formed.

In our enzyme-inhibitor complex structure, the lactam ring of N3 molecule serves as a structural analog to glutamine or histidine at P1 position (Fig. 2a), and protrudes into the S1 pocket by forming a 2.5 Å hydrogen bond between the lactam oxygen and the imidazole ring NH of His163 (Fig. 2b,c), which is similar to those in the complex structures of SARS-CoV M^pro^ and HCoV-HKU1 M^pro^ with inhibitor N3^49^,^53^. Furthermore, the NH of N3 lactam ring forms two hydrogen bonds with Oε1 of Glu166 (3.1 Å) and the carbonyl oxygen atom of Phe139 (3.2 Å) respectively, which provide additional support to stabilize the lactam ring in S1 pocket. The backbone NH of P1 residue on N3 also forms a 3.0 Å hydrogen bond with the backbone carbonyl oxygen from Gln164, favorably accommodating the S1 pocket.

### S2 pocket

The P2 position of natural M^pro^ cleavage sites across of the genomes of six human CoVs usually prefers a hydrophobic residue (Table 3). It is surprising that the P2 residues are completely identical among all 11 cleavage sites between the two alphacoronaviruses, HCoV-NL63 and HCoV-229E. In a majority of cases, it is leucine residue occupying the P2 position, with valine at nsp6-nsp7 and isoleucine at nsp10-nsp11. On the other hand, more variations are observed in the P2 position among betacoronaviruses, with more alternative residues such as methionine, phenylalanine, and proline, which might indicate less stringency of P2 specificity. The P2 site of inhibitor N3 mimics the side chain of leucine, in order to cover the maximum spectrum of natural M^pro^ substrates for human CoVs^49^. As observed in the HCoV-NL63 structure with N3, the aliphatic isobutyl side chain of P2 protrudes into the deep S2 pocket via interactions with the alkyl portion of the side chains of Asp187, Pro189, and is well accommodated onto the Van der Waals surface of the pocket (Fig. 2b).

**Figure 4:**
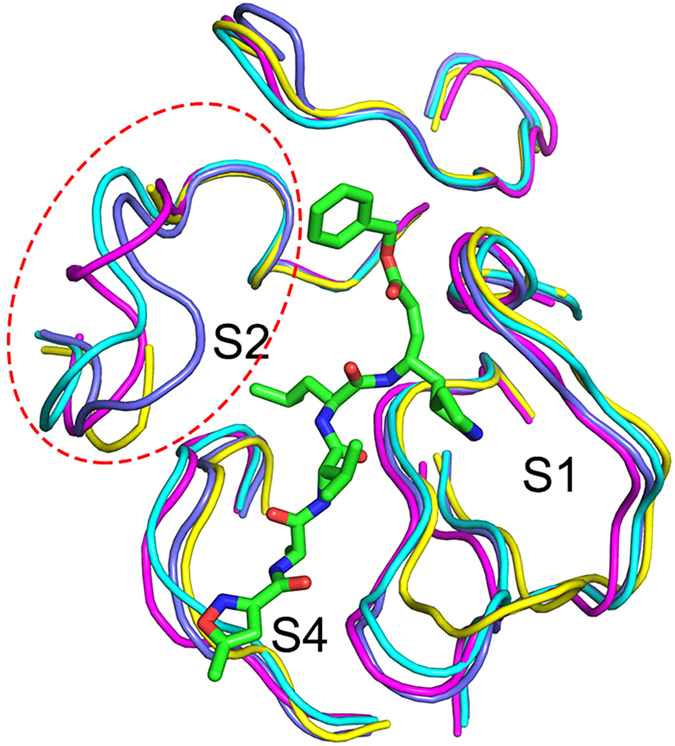
S1 and S2 binding sites in HCoV-NL63 M^pro^. The main chains of four human CoV M^pro^ structures (HCoV-NL63: slate, HCoV-229E: cyan, SARS-CoV: magenta, and HCoV-HKU1: yellow) are superimposed and displayed in the neightborhood of the substrate-binding site. The S1, S2, and S4 binding sites are labeled. The backbones are represented in worm form, and inhibitor is shown in stick format of green color. Residues 45–51 are marked with a red oval (in dash line).

To determine the structural diversity in S2 pocket, we then superimposed the backbones of four know M^pro^ structures from human CoVs. The lid of the S2 pocket in HCoV-NL63 is covered by a tight loop of residues 45–51 (Fig. 4). Interestingly, the same region in SARS-CoV and HCoV-HKU1 adopts a secondary structure of 3_10_ helix to maintain S2 pocket^53^. Such difference might partially account for the increased variation of natural P2 residues as observed in the genomes of betacoronaviruses.

### P3, P4, and P5 positions

The P3 side chains of N3 inhibitor are both solvent exposed in the two protomers of HCoV-NL63 M^pro^ (Figs 2b and 3b). This is consistent with the fact that no specificity for any particular side chains exists at the P3 position of cleavage sites among CoV M^pro^s^55^. Further, the NH and carbonyl oxygen of P3 backbone form two hydrogen bonds with the backbone carbonyl oxygen and NH of Glu166 respectively, which help anchor the N3 inhibitor to the HCoV-NL63 M^pro^ at P3 location.

The P4 position of the inhibitor N3 is alanine, and its side chain readily inserts into the relatively shallow P4 pocket (Fig. 3b), forming hydrophobic interactions with Pro189. Also the backbone NH of alanine residue on N3 donates a 2.9-Å hydrogen bond to the carbonyl oxygen of Ser190 (Fig. 2c). The isoxazole at P5 makes Van der Waals interactions with Gly168 and the backbone of residues Leu191 (Fig. 2b). Overall, the pattern of interactions between N3 and HCoV-NL63 M^pro^ at P3, P4, and P5 positions is similar to that between N3 and SARS-CoV M^pro ^49.

### Structural conservation of M^pro^ among clinical HCoV-NL63 isolates

**Figure 5:**
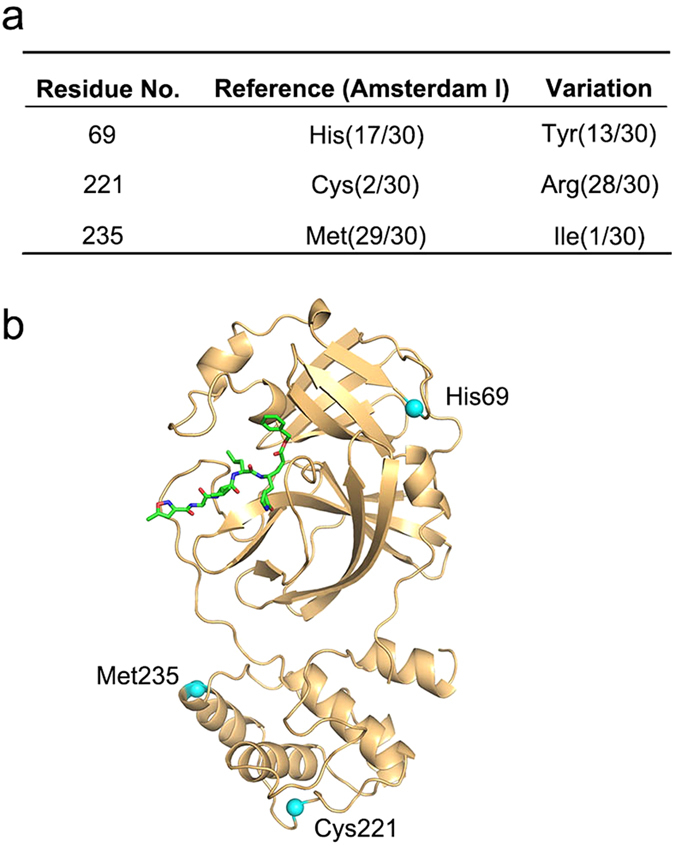
Structural conservation of M^pro^ among clinical HCoV-NL63 isolates. (**a**) Summary of the 3 residues of variation among a total of 30 HCoV-NL63 strains. Amsterdam I is used as reference strain. Number of strains carrying one particular residue is labeled in bracket. (**b**) Three-dimensional representation of the 3 nonconserved residues from (**a**) mapped onto HCoV-NL63 M^pro^ complex structure with inhibitor N3. Conserved residues are colored light orange, the 3 residues (His69, Cys221, and Met235) are colored cyan, and inhibitor N3 is colored green.

So far, whole genomic sequences of as many as 30 strains of HCoV-NL63 have been deposited into NCBI database^16^,^44^,^45^,^60^,^61^. These strains were isolated from patient specimens dated back to the past three decades from several countries, including The Netherlands, United States, and China. High percentage of sequence variation among these clinically isolated HCoV-NL63 strains and evidence of *in vivo* recombination during co-infections with other CoVs have been documented, especially in the N-terminal domain of spike protein and nsp2/nsp3 region^44^,^60^. In order to determine the efficacy of inhibitor N3 against sequence variation accumulated during the circulation of HCoV-NL63 in human population, we assessed the level of sequence and structural conservation in the molecular target of this inhibitor among different clinical isolates. Sequence for M^pro^ (nsp5) was retrieved from these HCoV-NL63 strains with available genomic sequences (Supplementary Table S1), and alignment was performed to determine sequence variations among these clinical isolates. M^pro^ sequence from strain Amsterdam I (NC_005831) was used as reference^16^,^44^. Overall, M^pro^ is extremely conserved among clinical HCoV-NL63 strains isolated worldwide (Supplementary Fig. S1), evidencing the enzyme’s essential role in viral replication. Sequence variation is only observed in 3 residues: His69 is mutated to Tyr in 13 strains isolated from United States, Cys221 is mutated to Arg among all the strains that have not been cultured *in vitro*, and Met235 is mutated to Ile in only one strain isolated in United States (Fig. 5a). When plotted onto the structure of HCoV-NL63 M^pro^, these 3 residues are all located in flexible regions, such as loop and molecular surface (Fig. 5b). None of the 3 residues are directly interacting with inhibitor N3, which provides clear structural evidence to support the effectiveness of N3 against all the clinical strains of HCoV-NL63, in spite of significant genomic sequence variation and potential for recombination in viral transmission.

### Inhibitor N3 targets a conserved site among human CoVs and their related zoonotic counterparts

**Figure 6:**
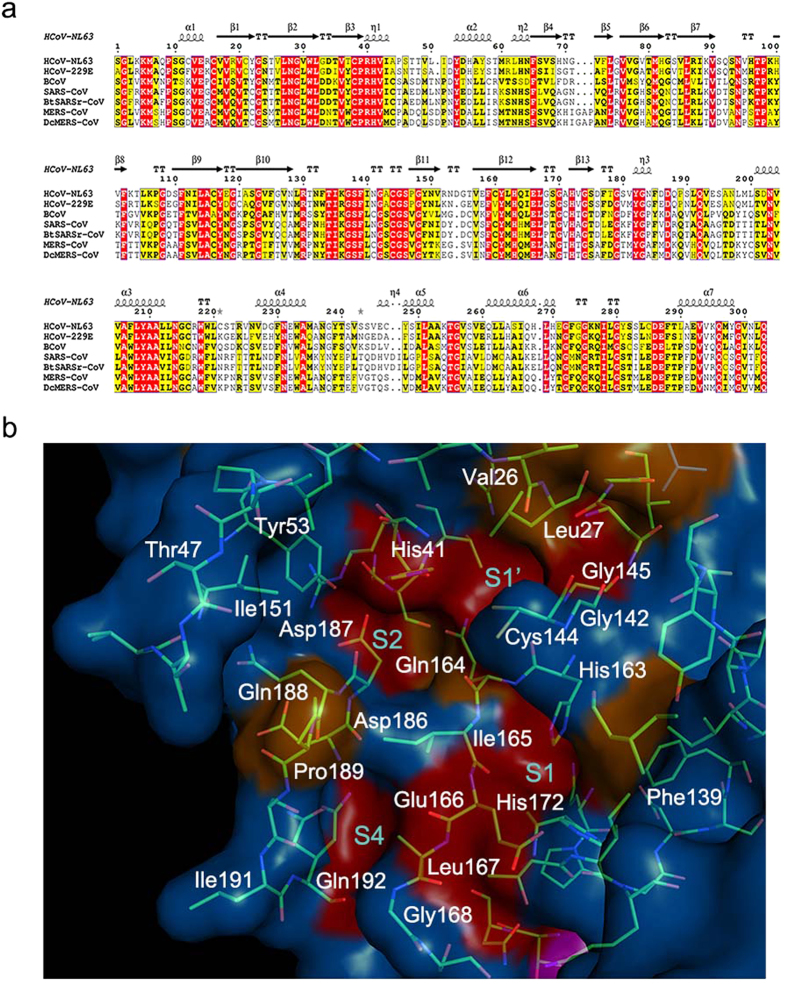
Inhibitor N3 targets a conserved site among human and related zoonotic CoVs. (**a**) Sequence alignment of three pairs of human CoVs and their related zoonotic counterparts: HCoV-229E and bovine coronavirus (BCoV), SARS-CoV and SARS-related coronavirus isolated from bat (BtSARSr-CoV), MERS-CoV and dromedary camel MERS-CoV (DcMERS-CoV). HCoV-NL63 and its secondary structure are used as reference for the alignment. Sequence alignment was performed using ClustalW2^72^, and figure was generated using ESPript 3.0^73^. (**b**) Surface representation of conserved substrate-binding pockets from 7 CoV M^pro^s listed in (a). Background is HCoV-NL63 M^pro^. Red: identical residues among all seven CoV M^pro^; magenta: substitution in one CoV M^pro^; orange: substitution in two CoV M^pro^s. The residues forming the substrate-binding pocket are labeled. S1’: Leu27, Gly142; S1: Phe139, His163, Glu166, His172; S2: His41 Tyr53, Asp187; S4: Leu167, Gln192.

The substrate-binding site of M^pro^ has been shown as a conserved drug target for designing wide spectrum anti-CoV inhibitors^48^,^49^,^51^,^53^. Given the evidence of repetitive global epidemics (such as SARS and MERS) caused by zoonotic transmission^11^,^19^,^20^,^21^,^22^,^23^,^24^, it is imperative to examine whether the substrate-binding site and the inhibition mechanism employed by inhibitor N3 are conserved between known human CoVs and their related zoonotic CoVs. We chose three most representative pairs of human CoVs and their zoonotic counterparts: HCoV-229E (NC_002645) and bovine coronavirus (BCoV, NC_003045), SARS-CoV (NC_004718) and SARS-related CoV isolated from bat (BtSARSr-CoV, KC881006), MERS-CoV (JX869059) and dromedary camel MERS-CoV (DcMERS-CoV, KJ713296). We then retrieved their M^pro^ sequences from NCBI database. The direct bat ancestor of HCoV-NL63 has not been identified^47^, therefore we only use HCoV-NL63 M^pro^ sequence and its secondary structure presented in this study as reference. Sequence alignment, shown in Fig. 6a, demonstrates significant homology in primary amino acid sequence among these 7 CoVs.

A closer examination at the substrate-binding pocket further reveals a conservative 3D architecture at this drug target (Fig. 6b). Those residues, which are critical for pocket formation, such as Leu27, Gly142 for pocket S1’; Phe139, His163, Glu166, His172 for pocket S1; His41, Tyr53, Asp187 for pocket S2; Leu167, Gln192 for pocket S4, are strictly conserved among various CoVs (Fig. 6a,b). Peptidomimetic inhibitor N3, through its Michael acceptor and well-designed side chains, snugly fits into the conserved substrate-binding pocket. The binding of N3 establishes a concerted interaction network, including a 1.8-Å covalent bond between Cβ of vinyl group on inhibitor N3 and the Sγ atom of Cys144, 7 hydrogen bonds and extensive hydrophobic interactions between N3 and the above residues critical for interplay. These findings demonstrate that inhibitor N3 could exert inhibitory effect towards a conserved site in CoVs both before and after zoonotic transmission. Therefore, in addition to its role in inhibiting known circulating human CoV species, inhibitor N3 might also serve as a lead compound for preclinical and clinical testing against potential future epidemic caused by CoV emerging from zoonotic origin.

## Discussion

The world has experienced two global outbreaks of CoV infections since entering the 21^st^ century^5^,^6^,^7^,^8^,^9^,^11^,^12^,^13^. Both SARS-CoV and MERS-CoV cause severe respiratory syndrome with high mortality rate^10^,^14^,^15^. In addition, four more human CoVs, namely HCoV-NL63, HCoV-229E, HCoV-HKU1, and HCoV-OC43, have been identified as pathological agents for common cold^9^,^16^,^17^. The lack of effective therapeutic and preventive strategies against human CoVs calls for immediate action of the scientific community^9^,^50^. We previously demonstrated that designing wide spectrum inhibitor at a conservative target is a viable method to develop anti-CoV therapeutics, given the high mutation and recombination rates observed in viral replication^48^,^49^,^51^,^53^. The ability of CoV to cross animal-human boundary provides further support to our strategy. Indeed, several human CoVs, including HCoV-NL63, SARS-CoV, and MERS-CoV, have been linked to zoonotic CoVs which naturally infect hosts such as bats or dromedary camels^20^,^21^,^22^,^23^,^24^,^25^,^46^. In current study, we use the M^pro^ from HCoV-NL63 as our model molecule, and present its crystal structure in complex with an inhibitor. Based on the structural detail at 2.85 Å resolution, the substrate-binding pocket of HCoV-NL63 M^pro^ is conserved among three pairs of human and zoonotic CoVs (Fig. 6). Several key residues at the subsites for substrate binding are completely identical among these seven coronaviruses, for example Phe139, His163, Glu166, His172 of S1; His41, Tyr53, Asp187 of S2; and Leu167, Gln192 of S4. These findings are consistent with the role of M^pro^ in viral replication, which is essential to the proteolytic processing and maturation of replicase polyprotein (encoded by ORF1). Analysis of substrate recognition sequence, based on available whole genome sequences of all six human CoVs, provides additional evidence to the conservation of M^pro^ substrate-binding pocket: the P1 position requires almost exclusively glutamine, and the P2 position exhibits strong preference for hydrophobic residues such as Leu and Val (Table 3). Taken together, through designing of wide spectrum inhibitors at a conservative site on M^pro^, our study outlines a novel therapeutic approach of containing diseases caused by both existing and possible future emerging human CoVs.

A close examination of the interaction between HCoV-NL63 M^pro^ and inhibitor N3 reveals structural details of inhibition mechanism. N3 is a synthetic peptidomimetic compound with Michael acceptor (Fig. 2a), which achieves mechanism-based enzyme inactivation through forming an irreversible covalent bond with Cys144 of catalytic dyad (Fig. 2b,c). The success of two serine protease inhibitors, telaprevir and boceprevir, in the treatment of hepatitis C virus (HCV) has underscored the importance of covalent inhibitors for targeting viral proteases^62^. Both telaprevir and boceprevir are peptidomimetic inhibitors carry a warhead of α-ketoamide, which forms a covalent yet reversible bond with catalytic triad serine residue of HCV NS3∙4 A protease^62^,^63^. Michael acceptor has also been used as a warhead in pharmaceutical targeting against viral protease, for example rupintrivir was developed as an inhibitor for 3C protease of human rhinovirus and enterovirus^50^,^64^,^65^. The backbone of N3 forms 7 hydrogen bonds with residues in the substrate-binding pocket (Fig. 2c). The pockets accommodating N3 undergo gate-regulated switch to facilitate the binding of inhibitor N3 (Fig. 3), an interesting phenomena initially observed in case of M^pro^ from SARS-CoV^49^.

In addition, the structure of HCoV-NL63 M^pro^ exhibits several unique but interesting features. Examination of its natural cleavage sites based on HCoV-NL63 genomic sequence reveals an unusual histidine P1 residue between nsp13 and nsp14, which is unique to HCoV-NL63 and HCoV-HKU1 among all human CoVs^37^. Such P1 anomaly is partially accommodated by the relatively smaller S1 pocket, as the size of its S1 pocket is comparable to that of HCoV-HKU1, but smaller than those of HCoV-229E and SARS-CoV. The P2 position seems to present genus specificity among alphacoronaviruses, as the P2 residues among all 11 natural cleavage sites are completely identical between HCoV-NL63 and HCoV-229E with a strong dominance of leucine. While large variation on P2 residue is observed in the genomes of human betacoronaviruses.

In summary, the crystal structure of HCoV-NL63 M^pro^ complexed with inhibitor N3 has provided critical insight into the design of irreversible inhibitor carrying a Michael acceptor warhead. Through detailed sequence and structural comparison, this compound demonstrates feasibility as potential broad spectrum therapeutic agent for both existing and possibly emerging human CoVs. Further pharmaceutical development of such covalent peptidomimetic inhibitors would yield success in clinical management of human coronavirus diseases and public health preparedness for possible future pandemic.

## Methods

### Protein expression and purification

Protein expression and purification of HCoV-NL63 main protease has been described previously^66^. The coding sequence was subcloned into pGEX-6P-1 vector, and transformed into BL21 (DE3) *E. coli* cells. Cell culture was first grown in LB medium containing 100 μg ml^−1^ ampicillin at 37 °C until optical density (OD600) reached 0.6. Protein expression was then induced by adding isopropyl β-D-1-thiogalactopyranoside to a final concentration of 0.5 mM and further cultured at 16 °C for 16 hours. Cell pellets were harvested by centrifugation, and resuspended in phosphate-buffered saline solution supplemented with 1 mM dithiothreitol (DTT) and 10% glycerol. Cell lysate was prepared using sonication and centrifugation (12,000 g, 50 min, 4 °C). GST-tagged HCoV-NL63 M^pro^ fusion protein was bound to glutathione sepharose 4B affinity resin, and GST tag was removed through on-column cleavage using commercial PreScission protease (GE Healthcare) at 4 °C for 18 hours. Recombinant HCoV-NL63 M^pro^ protein was subject to an additional step of anion-exchange chromatography using HiTrap Q column (GE Healthcare), and was eluted with a linear gradient of 25 to 250 mM NaCl (20 mM Tris-HCl pH = 8.0, 10% glycerol, 1 mM DTT).

### Crystallization and data collection

Purified protein was supplemented with 10% DMSO and concentrated to 1 mg ml^−1^. Crystals of HCoV-NL63 M^pro^ in complex with inhibitor N3 were produced by cocrystallization. Inhibitor N3 was added to HCoV M^pro^ protein at a molar ratio between 3:1 and 5:1, and incubated at 4 °C for 4 hours. The complex was then centrifuged at 12,000 g for 10 min, and concentrated to 10 mg ml^−1^ in a buffer containing 10 mM HEPES pH 7.5, 150 mM NaCl, 1 mM DTT. Using hanging-drop vapor diffusion method at 16 °C, best crystals were obtained after 2 days using a reservoir solution containing 0.1 M HEPES pH 5.5, 10%(w/v) polyethylene glycol 8000, 4%(v/v) ethylene glycol (PDB entry 3TLO) and 0.1 M sodium citrate tribasic dehydrate pH 5.6, 1.0 M ammonium phosphate monobasic in a ratio of 80:20^67^.

Data for HCoV-NL63 M^pro^-N3 complex was collected to a 2.85-Å resolution at 100 K on beamline 1W2B of Beijing Synchrotron Radiation Facility (BSRF), using a MAR165 charge-coupled device detector. The cryoprotectant solution contained 20% (v/v) glycerol, 10% (w/v) polyethylene glycol 8000, 4% (v/v) ethylene glycol, and 0.1 M HEPES pH 5.5. All data integration and scaling were performed using HKL2000^68^. The Matthews coefficient of the crystal suggested two molecules per asymmetric unit, and the solvent content was 59.8%.

### Structure determination and analysis

The structure of HCoV-NL63 M^pro^-N3 complex was determined using molecular replacement from that of apo-form HCoV-NL63 M^pro^ (PDB ID: 3TLO; C. P. Chuck & K. B. Wong, unpublished work). All cross-rotation and translation searches for molecular replacement were performed with Phaser^69^. Cycles of manual adjustment using Coot^70^ and subsequent refinement using PHENIX^71^ led to a final model with a crystallographic R factor (R_cryst_) of 19.4% and a free R factor (R_free_) of 24.1% at 2.85-Å resolution.

### Enzymatic activity and inhibition assays

Enzymatic assay was performed using a fluorogenic substrate with consensus sequence of CoV M^pro^, MCA-AVLQSGFR-Lys(Dnp)-Lys-NH2 (>95% purity, GL Biochem Shanghai Ltd., Shanghai, China), as previously reported^48^,^49^,^53^. Fluorescence intensity was monitored using a Fluoroskan Ascent instrument (Thermo Scientific, USA) with excitation and emission wavelengths of 320 nm and 405 nm, respectively. The assay was performed in a buffer solution consisted of 50 mM Tris-HCl (pH 7.3) and 1 mM EDTA at 30 °C. Kinetic parameters, including *K*_*m*_ and *k*_*cat*_ of apo HCoV-NL63 M^pro^ and *K*_*i*_ and *k*_*3*_ of inhibitor N3, were determined using methods described in detail in our previous work^49^.

## Additional Information

**How to cite this article**: Wang, F. *et al.* Structure of Main Protease from Human Coronavirus NL63: Insights for Wide Spectrum Anti-Coronavirus Drug Design. *Sci. Rep.* **6**, 22677; doi: 10.1038/srep22677 (2016).

## Supplementary Material

Table S1, Figure S1

## Figures and Tables

**Table 1:** X-ray data-processing and refinement statistics.

Statistics	Value for the HCoV-NL63 M^pro^-N3 complex
Data collection	
Wavelength (Å)	1.0000
Resolution limit (Å)	50.0–2.85 (2.90–2.85)
Space group	*P*4_1_2_1_2
Cell parameters	
a (Å)	87.2
b (Å)	87.2
c (Å)	212.1
α = β = γ (°)	90
Total no. of reflections	283898
No. of unique reflections	19967
Completeness (%)	100 (100)
Redundancy	14.2 (12.6)
R_merge_ (%)	14.4 (46.6)
Sigma cutoff	0
*I/σ(I)*	21.5 (6.1)
Refinement	
Resolution range (Å)	50.0–2.85
R_work_ (%)	19.3
R_free_ (%)	24.1
Rmsd from ideal geometry	
Bonds (Å)	0.007
Angles (°)	1.16
Avg B factor (Å^2^)	27.6
Protein	28.1
Small molecule	28.3
Ramachandran plot	
Favored (%)	96.0
Allowed (%)	4.0
Outliers (%)	0.0

**Table 2:** Enzyme activity and N3 inhibition data for HCoV-NL63 M^pro^.

Virus M^pro^	*K*_*m*_ (μM)	*k*_*cat*_ (s^−1^)	Inhibitor N3		Data Source
			*K*_*i*_ (μM)	*k*_*3*_ (10^−3^∙s^−1^)	
HCoV-NL63	50.8 ± 3.4	0.098 ± 0.004	11.3 ± 1.0	42.4 ± 5.0	This study
SARS-CoV	129 ± 7	0.14 ± 0.01	9.0 ± 0.8	3.1 ± 0.5	49
HCoV-229E	29.8 ± 0.9	1.27 ± 0.09	1.67 ± 0.18	18.0 ± 1.1	49
HCoV-HKU1	83.2 ± 13.3	1.1 ± 0.12	−	−	53

**Table 3:** P2 residues from genomes of six human CoVs.

No.	Cleavage Site	Alphacoronavirus		Betacoronavirus			
		HCoV-NL63	HCoV-229E	HCoV-HKU1	HCoV-OC43	SARS-CoV	MERS-CoV
1	nsp4-nsp5	L	L	L	L	L	L
2	nsp5-nsp6	L	L	L	L	F	M
3	nsp6-nsp7	V	V	I	F	V	M
4	nsp7-nsp8	L	L	L	L	L	L
5	nsp8-nsp9	L	L	M	L	L	L
6	nsp9-nsp10	L	L	L	L	L	L
7	nsp10-nsp11	I	I	V	V	M	P
8	nsp12-nsp13	L	L	M	M	L	L
9	nsp13-nsp14	L	L	L	V	L	L
10	nsp14-nsp15	L	L	L	L	L	V
11	nsp15-nsp16	L	L	M	L	L	L
