# Supplementary material for: Structure of Main Protease from Human Coronavirus NL63: Insights for Wide Spectrum Anti-Coronavirus Drug Design
Source: Sci Rep. 2016 Mar 7;6:22677. doi: 10.1038/srep22677 (PMC4780191; doi:10.1038/srep22677)
Supplement: Table S1, Figure S1 [file srep22677-s1.pdf]

Structure of Main Protease from Human Coronavirus NL63: Insights for Wide Spectrum Anti-Coronavirus Drug Design

Fenghua Wang[1,+], Cheng Chen[1,2,+], Wenjie Tan[3], Kailin Yang[4,*], Haitao Yang[1,2,*]

[1]School of Life Sciences, Tianjin University, Tianjin, China.

[2]Tianjin International Joint Academy of Biotechnology and Medicine, Tianjin, China.

[3]Key Laboratory of Medical Virology, Ministry of Health, National Institute for Viral Disease Control and Prevention, Chinese Center for Disease Control and Prevention, Beijing, China.

[4]Cleveland Clinic Lerner College of Medicine of Case Western Reserve University, Cleveland, OH, USA.

[+]These authors contributed equally to this work.

*Correspondence and requests for materials should be addressed to H.Y. (email: yanght@tju.edu.cn) or K.Y. (email: yangk2@ccf.org)

Table S1. Name, NCBI accession number, and country of isolation of 30 NL63 clinical strains with complete genome sequence available.

No.	Strain name	NCBI accession number	Country of isolation
1	Amsterdam I	NC_005831	The Netherlands
2	Amsterdam 057	DQ445911	The Netherlands
3	Amsterdam 496	DQ445912	The Netherlands
4	CBJ 037	JX104161	China
5	CBJ 123	JX524171	China
6	RPTEC/2004	JX504050	United States
7	USA/838-9/1983	KF530110	United States
8	USA/8712-17/1987	KF530106	United States
9	USA/891-4/1989	KF530114	United States
10	USA/891-6/1989	KF530108	United States
11	USA/901-24/1990	KF530111	United States
12	USA/903-28/1990	KF530109	United States
13	USA/904-20/1990	KF530104	United States
14	USA/905-25/1990	KF530113	United States
15	USA/911-56/1991	KF530107	United States
16	USA/0111-25/2001	KF530112	United States
17	USA/012-31/2001	KF530105	United States
18	DEN/2005/193	JQ765568	United States
19	DEN/2005/232	JQ765569	United States
20	DEN/2005/235	JQ765570	United States
21	DEN/2005/271	JQ765571	United States
22	DEN/2005/347	JQ765572	United States
23	DEN/2005/1062	JQ765573	United States
24	DEN/2005/1862	JQ765574	United States
25	DEN/2005/1876	JQ765575	United States
26	DEN/2009/9	JQ765563	United States
27	DEN/2009/14	JQ765564	United States
28	DEN/2009/15	JQ765565	United States
29	DEN/2008/16	JQ765566	United States
30	DEN/2009/20	JQ765567	United States

Figure S1. Sequence alignment of M^{pro} from 30 NL63 clinical strains listed in Table S1. Sequence alignment was performed using ClustalW2, and figure was generated using ESPript 3.0.

	1 10 20 30 40 50 60 70
NC_005831_Amsterdam-I	SGLKKMAQPSGCVERCVVRVCYGSTVLNGVWLGDTVTCPRHVIAPSTTVLIDYDHAYSTMRLHNFSVSHN
DQ445911_Amsterdam-057	SGLKKMAQPSGCVERCVVRVCYGSTVLNGVWLGDTVTCPRHVIAPSTTVLIDYDHAYSTMRLHNFSVSHN
DQ445912_Amsterdam-496	SGLKKMAQPSGCVERCVVRVCYGSTVLNGVWLGDTVTCPRHVIAPSTTVLIDYDHAYSTMRLHNFSVSHN
JX104161_CBJ-037	SGLKKMAQPSGCVERCVVRVCYGSTVLNGVWLGDTVTCPRHVIAPSTTVLIDYDHAYSTMRLHNFSVSHN
JX524171_CBJ-123	SGLKKMAQPSGCVERCVVRVCYGSTVLNGVWLGDTVTCPRHVIAPSTTVLIDYDHAYSTMRLHNFSVSHN
JX504050_RPTEC/2004	SGLKKMAQPSGCVERCVVRVCYGSTVLNGVWLGDTVTCPRHVIAPSTTVLIDYDHAYSTMRLHNFSVSHN
KF530110_USA/838-9/1983	SGLKKMAQPSGCVERCVVRVCYGSTVLNGVWLGDTVTCPRHVIAPSTTVLIDYDHAYSTMRLHNFSVSYN
KF530106_USA/8712-17/1987	SGLKKMAQPSGCVERCVVRVCYGSTVLNGVWLGDTVTCPRHVIAPSTTVLIDYDHAYSTMRLHNFSVSYN
KF530114_USA/891-4/1989	SGLKKMAQPSGCVERCVVRVCYGSTVLNGVWLGDTVTCPRHVIAPSTTVLIDYDHAYSTMRLHNFSVSYN
KF530108_USA/891-6/1989	SGLKKMAQPSGCVERCVVRVCYGSTVLNGVWLGDTVTCPRHVIAPSTTVLIDYDHAYSTMRLHNFSVSYN
KF530111_USA/901-24/1990	SGLKKMAQPSGCVERCVVRVCYGSTVLNGVWLGDTVTCPRHVIAPSTTVLIDYDHAYSTMRLHNFSVSYN
KF530109_USA/903-28/1990	SGLKKMAQPSGCVERCVVRVCYGSTVLNGVWLGDTVTCPRHVIAPSTTVLIDYDHAYSTMRLHNFSVSYN
KF530104_USA/904-20/1990	SGLKKMAQPSGCVERCVVRVCYGSTVLNGVWLGDTVTCPRHVIAPSTTVLIDYDHAYSTMRLHNFSVSYN
KF530113_USA/905-25/1990	SGLKKMAQPSGCVERCVVRVCYGSTVLNGVWLGDTVTCPRHVIAPSTTVLIDYDHAYSTMRLHNFSVSYN
KF530107_USA/911-56/1991	SGLKKMAQPSGCVERCVVRVCYGSTVLNGVWLGDTVTCPRHVIAPSTTVLIDYDHAYSTMRLHNFSVSYN
KF530112_USA/0111-25/2001	SGLKKMAQPSGCVERCVVRVCYGSTVLNGVWLGDTVTCPRHVIAPSTTVLIDYDHAYSTMRLHNFSVSHN
KF530105_USA/012-31/2001	SGLKKMAQPSGCVERCVVRVCYGSTVLNGVWLGDTVTCPRHVIAPSTTVLIDYDHAYSTMRLHNFSVSYN
JQ765568_DEN/2005/193	SGLKKMAQPSGCVERCVVRVCYGSTVLNGVWLGDTVTCPRHVIAPSTTVLIDYDHAYSTMRLHNFSVSHN
JQ765569_DEN/2005/232	SGLKKMAQPSGCVERCVVRVCYGSTVLNGVWLGDTVTCPRHVIAPSTTVLIDYDHAYSTMRLHNFSVSHN
JQ765570_DEN/2005/235	SGLKKMAQPSGCVERCVVRVCYGSTVLNGVWLGDTVTCPRHVIAPSTTVLIDYDHAYSTMRLHNFSVSHN
JQ765571_DEN/2005/271	SGLKKMAQPSGCVERCVVRVCYGSTVLNGVWLGDTVTCPRHVIAPSTTVLIDYDHAYSTMRLHNFSVSHN
JQ765572_DEN/2005/347	SGLKKMAQPSGCVERCVVRVCYGSTVLNGVWLGDTVTCPRHVIAPSTTVLIDYDHAYSTMRLHNFSVSHN
JQ765573_DEN/2005/1062	SGLKKMAQPSGCVERCVVRVCYGSTVLNGVWLGDTVTCPRHVIAPSTTVLIDYDHAYSTMRLHNFSVSHN
JQ765574_DEN/2005/1862	SGLKKMAQPSGCVERCVVRVCYGSTVLNGVWLGDTVTCPRHVIAPSTTVLIDYDHAYSTMRLHNFSVSYN
JQ765575_DEN/2005/1876	SGLKKMAQPSGCVERCVVRVCYGSTVLNGVWLGDTVTCPRHVIAPSTTVLIDYDHAYSTMRLHNFSVSHN
JQ765563_DEN/2009/9	SGLKKMAQPSGCVERCVVRVCYGSTVLNGVWLGDTVTCPRHVIAPSTTVLIDYDHAYSTMRLHNFSVSHN
JQ765564_DEN/2009/14	SGLKKMAQPSGCVERCVVRVCYGSTVLNGVWLGDTVTCPRHVIAPSTTVLIDYDHAYSTMRLHNFSVSHN
JQ765565_DEN/2009/15	SGLKKMAQPSGCVERCVVRVCYGSTVLNGVWLGDTVTCPRHVIAPSTTVLIDYDHAYSTMRLHNFSVSHN
JQ765566_DEN/2008/16	SGLKKMAQPSGCVERCVVRVCYGSTVLNGVWLGDTVTCPRHVIAPSTTVLIDYDHAYSTMRLHNFSVSYN
JQ765567_DEN/2009/20	SGLKKMAQPSGCVERCVVRVCYGSTVLNGVWLGDTVTCPRHVIAPSTTVLIDYDHAYSTMRLHNFSVSYN

	80 90 100 110 120 130 140
NC_005831_Amsterdam-I	GVFLGVVGVTMHGSVLRIKVSQSNVHTPKHVFKTLKPGDSFNILACYEGIASGVFGVNLRTNFTIKGSFI
DQ445911_Amsterdam-057	GVFLGVVGVTMHGSVLRIKVSQSNVHTPKHVFKTLKPGDSFNILACYEGIASGVFGVNLRTNFTIKGSFI
DQ445912_Amsterdam-496	GVFLGVVGVTMHGSVLRIKVSQSNVHTPKHVFKTLKPGDSFNILACYEGIASGVFGVNLRTNFTIKGSFI
JX104161_CBJ-037	GVFLGVVGVTMHGSVLRIKVSQSNVHTPKHVFKTLKPGDSFNILACYEGIASGVFGVNLRTNFTIKGSFI
JX524171_CBJ-123	GVFLGVVGVTMHGSVLRIKVSQSNVHTPKHVFKTLKPGDSFNILACYEGIASGVFGVNLRTNFTIKGSFI
JX504050_RPTEC/2004	GVFLGVVGVTMHGSVLRIKVSQSNVHTPKHVFKTLKPGDSFNILACYEGIASGVFGVNLRTNFTIKGSFI
KF530110_USA/838-9/1983	GVFLGVVGVTMHGSVLRIKVSQSNVHTPKHVFKTLKPGDSFNILACYEGIASGVFGVNLRTNFTIKGSFI
KF530106_USA/8712-17/1987	GVFLGVVGVTMHGSVLRIKVSQSNVHTPKHVFKTLKPGDSFNILACYEGIASGVFGVNLRTNFTIKGSFI
KF530114_USA/891-4/1989	GVFLGVVGVTMHGSVLRIKVSQSNVHTPKHVFKTLKPGDSFNILACYEGIASGVFGVNLRTNFTIKGSFI
KF530108_USA/891-6/1989	GVFLGVVGVTMHGSVLRIKVSQSNVHTPKHVFKTLKPGDSFNILACYEGIASGVFGVNLRTNFTIKGSFI
KF530111_USA/901-24/1990	GVFLGVVGVTMHGSVLRIKVSQSNVHTPKHVFKTLKPGDSFNILACYEGIASGVFGVNLRTNFTIKGSFI
KF530109_USA/903-28/1990	GVFLGVVGVTMHGSVLRIKVSQSNVHTPKHVFKTLKPGDSFNILACYEGIASGVFGVNLRTNFTIKGSFI
KF530104_USA/904-20/1990	GVFLGVVGVTMHGSVLRIKVSQSNVHTPKHVFKTLKPGDSFNILACYEGIASGVFGVNLRTNFTIKGSFI
KF530113_USA/905-25/1990	GVFLGVVGVTMHGSVLRIKVSQSNVHTPKHVFKTLKPGDSFNILACYEGIASGVFGVNLRTNFTIKGSFI
KF530107_USA/911-56/1991	GVFLGVVGVTMHGSVLRIKVSQSNVHTPKHVFKTLKPGDSFNILACYEGIASGVFGVNLRTNFTIKGSFI
KF530112_USA/0111-25/2001	GVFLGVVGVTMHGSVLRIKVSQSNVHTPKHVFKTLKPGDSFNILACYEGIASGVFGVNLRTNFTIKGSFI
KF530105_USA/012-31/2001	GVFLGVVGVTMHGSVLRIKVSQSNVHTPKHVFKTLKPGDSFNILACYEGIASGVFGVNLRTNFTIKGSFI
JQ765568_DEN/2005/193	GVFLGVVGVTMHGSVLRIKVSQSNVHTPKHVFKTLKPGDSFNILACYEGIASGVFGVNLRTNFTIKGSFI
JQ765569_DEN/2005/232	GVFLGVVGVTMHGSVLRIKVSQSNVHTPKHVFKTLKPGDSFNILACYEGIASGVFGVNLRTNFTIKGSFI
JQ765570_DEN/2005/235	GVFLGVVGVTMHGSVLRIKVSQSNVHTPKHVFKTLKPGDSFNILACYEGIASGVFGVNLRTNFTIKGSFI
JQ765571_DEN/2005/271	GVFLGVVGVTMHGSVLRIKVSQSNVHTPKHVFKTLKPGDSFNILACYEGIASGVFGVNLRTNFTIKGSFI
JQ765572_DEN/2005/347	GVFLGVVGVTMHGSVLRIKVSQSNVHTPKHVFKTLKPGDSFNILACYEGIASGVFGVNLRTNFTIKGSFI
JQ765573_DEN/2005/1062	GVFLGVVGVTMHGSVLRIKVSQSNVHTPKHVFKTLKPGDSFNILACYEGIASGVFGVNLRTNFTIKGSFI
JQ765574_DEN/2005/1862	GVFLGVVGVTMHGSVLRIKVSQSNVHTPKHVFKTLKPGDSFNILACYEGIASGVFGVNLRTNFTIKGSFI
JQ765575_DEN/2005/1876	GVFLGVVGVTMHGSVLRIKVSQSNVHTPKHVFKTLKPGDSFNILACYEGIASGVFGVNLRTNFTIKGSFI
JQ765563_DEN/2009/9	GVFLGVVGVTMHGSVLRIKVSQSNVHTPKHVFKTLKPGDSFNILACYEGIASGVFGVNLRTNFTIKGSFI
JQ765564_DEN/2009/14	GVFLGVVGVTMHGSVLRIKVSQSNVHTPKHVFKTLKPGDSFNILACYEGIASGVFGVNLRTNFTIKGSFI
JQ765565_DEN/2009/15	GVFLGVVGVTMHGSVLRIKVSQSNVHTPKHVFKTLKPGDSFNILACYEGIASGVFGVNLRTNFTIKGSFI
JQ765566_DEN/2008/16	GVFLGVVGVTMHGSVLRIKVSQSNVHTPKHVFKTLKPGDSFNILACYEGIASGVFGVNLRTNFTIKGSFI
JQ765567_DEN/2009/20	GVFLGVVGVTMHGSVLRIKVSQSNVHTPKHVFKTLKPGDSFNILACYEGIASGVFGVNLRTNFTIKGSFI

```
                                       150       160       170       180       190       200       210
NC_005831_Amsterdam-I          NGACGSPGYNVRNDGTVEFCYLHQIELGSGAHVGSDFTGSVYGNFDDQPSLQVESANLMLSDNVVAFLYA
DQ445911_Amsterdam-057         NGACGSPGYNVRNDGTVEFCYLHQIELGSGAHVGSDFTGSVYGNFDDQPSLQVESANLMLSDNVVAFLYA
DQ445912_Amsterdam-496         NGACGSPGYNVRNDGTVEFCYLHQIELGSGAHVGSDFTGSVYGNFDDQPSLQVESANLMLSDNVVAFLYA
JX104161_CBJ-037               NGACGSPGYNVRNDGTVEFCYLHQIELGSGAHVGSDFTGSVYGNFDDQPSLQVESANLMLSDNVVAFLYA
JX524171_CBJ-123               NGACGSPGYNVRNDGTVEFCYLHQIELGSGAHVGSDFTGSVYGNFDDQPSLQVESANLMLSDNVVAFLYA
JX504050_RPTEC/2004            NGACGSPGYNVRNDGTVEFCYLHQIELGSGAHVGSDFTGSVYGNFDDQPSLQVESANLMLSDNVVAFLYA
KF530110_USA/838-9/1983        NGACGSPGYNVRNDGTVEFCYLHQIELGSGAHVGSDFTGSVYGNFDDQPSLQVESANLMLSDNVVAFLYA
KF530106_USA/8712-17/1987      NGACGSPGYNVRNDGTVEFCYLHQIELGSGAHVGSDFTGSVYGNFDDQPSLQVESANLMLSDNVVAFLYA
KF530114_USA/891-4/1989        NGACGSPGYNVRNDGTVEFCYLHQIELGSGAHVGSDFTGSVYGNFDDQPSLQVESANLMLSDNVVAFLYA
KF530108_USA/891-6/1989        NGACGSPGYNVRNDGTVEFCYLHQIELGSGAHVGSDFTGSVYGNFDDQPSLQVESANLMLSDNVVAFLYA
KF530111_USA/901-24/1990       NGACGSPGYNVRNDGTVEFCYLHQIELGSGAHVGSDFTGSVYGNFDDQPSLQVESANLMLSDNVVAFLYA
KF530109_USA/903-28/1990       NGACGSPGYNVRNDGTVEFCYLHQIELGSGAHVGSDFTGSVYGNFDDQPSLQVESANLMLSDNVVAFLYA
KF530104_USA/904-20/1990       NGACGSPGYNVRNDGTVEFCYLHQIELGSGAHVGSDFTGSVYGNFDDQPSLQVESANLMLSDNVVAFLYA
KF530113_USA/905-25/1990       NGACGSPGYNVRNDGTVEFCYLHQIELGSGAHVGSDFTGSVYGNFDDQPSLQVESANLMLSDNVVAFLYA
KF530107_USA/911-56/1991       NGACGSPGYNVRNDGTVEFCYLHQIELGSGAHVGSDFTGSVYGNFDDQPSLQVESANLMLSDNVVAFLYA
KF530112_USA/0111-25/2001      NGACGSPGYNVRNDGTVEFCYLHQIELGSGAHVGSDFTGSVYGNFDDQPSLQVESANLMLSDNVVAFLYA
KF530105_USA/012-31/2001       NGACGSPGYNVRNDGTVEFCYLHQIELGSGAHVGSDFTGSVYGNFDDQPSLQVESANLMLSDNVVAFLYA
JQ765568_DEN/2005/193          NGACGSPGYNVRNDGTVEFCYLHQIELGSGAHVGSDFTGSVYGNFDDQPSLQVESANLMLSDNVVAFLYA
JQ765569_DEN/2005/232          NGACGSPGYNVRNDGTVEFCYLHQIELGSGAHVGSDFTGSVYGNFDDQPSLQVESANLMLSDNVVAFLYA
JQ765570_DEN/2005/235          NGACGSPGYNVRNDGTVEFCYLHQIELGSGAHVGSDFTGSVYGNFDDQPSLQVESANLMLSDNVVAFLYA
JQ765571_DEN/2005/271          NGACGSPGYNVRNDGTVEFCYLHQIELGSGAHVGSDFTGSVYGNFDDQPSLQVESANLMLSDNVVAFLYA
JQ765572_DEN/2005/347          NGACGSPGYNVRNDGTVEFCYLHQIELGSGAHVGSDFTGSVYGNFDDQPSLQVESANLMLSDNVVAFLYA
JQ765573_DEN/2005/1062         NGACGSPGYNVRNDGTVEFCYLHQIELGSGAHVGSDFTGSVYGNFDDQPSLQVESANLMLSDNVVAFLYA
JQ765574_DEN/2005/1862         NGACGSPGYNVRNDGTVEFCYLHQIELGSGAHVGSDFTGSVYGNFDDQPSLQVESANLMLSDNVVAFLYA
JQ765575_DEN/2005/1876         NGACGSPGYNVRNDGTVEFCYLHQIELGSGAHVGSDFTGSVYGNFDDQPSLQVESANLMLSDNVVAFLYA
JQ765563_DEN/2009/9            NGACGSPGYNVRNDGTVEFCYLHQIELGSGAHVGSDFTGSVYGNFDDQPSLQVESANLMLSDNVVAFLYA
JQ765564_DEN/2009/14           NGACGSPGYNVRNDGTVEFCYLHQIELGSGAHVGSDFTGSVYGNFDDQPSLQVESANLMLSDNVVAFLYA
JQ765565_DEN/2009/15           NGACGSPGYNVRNDGTVEFCYLHQIELGSGAHVGSDFTGSVYGNFDDQPSLQVESANLMLSDNVVAFLYA
JQ765566_DEN/2008/16           NGACGSPGYNVRNDGTVEFCYLHQIELGSGAHVGSDFTGSVYGNFDDQPSLQVESANLMLSDNVVAFLYA
JQ765567_DEN/2009/20           NGACGSPGYNVRNDGTVEFCYLHQIELGSGAHVGSDFTGSVYGNFDDQPSLQVESANLMLSDNVVAFLYA
```

```
                                       220       230       240       250       260       270       280
NC_005831_Amsterdam-I          ALLNGCRWWLCSTRVNVDGFNEWAMANGYTSVSSVECYSILAAKTGVSVEQLLASIQHLHEGFGGKNILG
DQ445911_Amsterdam-057         ALLNGCRWWLRSTRVNVDGFNEWAMANGYTSVSSVECYSILAAKTGVSVEQLLASIQHLHEGFGGKNILG
DQ445912_Amsterdam-496         ALLNGCRWWLRSTRVNVDGFNEWAMANGYTSVSSVECYSILAAKTGVSVEQLLASIQHLHEGFGGKNILG
JX104161_CBJ-037               ALLNGCRWWLRSTRVNVDGFNEWAMANGYTSVSSVECYSILAAKTGVSVEQLLASIQHLHEGFGGKNILG
JX524171_CBJ-123               ALLNGCRWWLRSTRVNVDGFNEWAMANGYTSVSSVECYSILAAKTGVSVEQLLASIQHLHEGFGGKNILG
JX504050_RPTEC/2004            ALLNGCRWWLCSTRVNVDGFNEWAMANGYTSVSSVECYSILAAKTGVSVEQLLASIQHLHEGFGGKNILG
KF530110_USA/838-9/1983        ALLNGCRWWLRSTRVNVDGFNEWAMANGYTSVSSVECYSILAAKTGVSVEQLLASIQHLHEGFGGKNILG
KF530106_USA/8712-17/1987      ALLNGCRWWLRSTRVNVDGFNEWAMANGYTSVSSVECYSILAAKTGVSVEQLLASIQHLHEGFGGKNILG
KF530114_USA/891-4/1989        ALLNGCRWWLRSTRVNVDGFNEWAMANGYTSVSSVECYSILAAKTGVSVEQLLASIQHLHEGFGGKNILG
KF530108_USA/891-6/1989        ALLNGCRWWLRSTRVNVDGFNEWAMANGYTSVSSVECYSILAAKTGVSVEQLLASIQHLHEGFGGKNILG
KF530111_USA/901-24/1990       ALLNGCRWWLRSTRVNVDGFNEWAMANGYTSVSSVECYSILAAKTGVSVEQLLASIQHLHEGFGGKNILG
KF530109_USA/903-28/1990       ALLNGCRWWLRSTRVNVDGFNEWAIANGYTSVSSVECYSILAAKTGVSVEQLLASIQHLHEGFGGKNILG
KF530104_USA/904-20/1990       ALLNGCRWWLRSTRVNVDGFNEWAMANGYTSVSSVECYSILAAKTGVSVEQLLASIQHLHEGFGGKNILG
KF530113_USA/905-25/1990       ALLNGCRWWLRSTRVNVDGFNEWAMANGYTSVSSVECYSILAAKTGVSVEQLLASIQHLHEGFGGKNILG
KF530107_USA/911-56/1991       ALLNGCRWWLRSTRVNVDGFNEWAMANGYTSVSSVECYSILAAKTGVSVEQLLASIQHLHEGFGGKNILG
KF530112_USA/0111-25/2001      ALLNGCRWWLRSTRVNVDGFNEWAMANGYTSVSSVECYSILAAKTGVSVEQLLASIQHLHEGFGGKNILG
KF530105_USA/012-31/2001       ALLNGCRWWLRSTRVNVDGFNEWAMANGYTSVSSVECYSILAAKTGVSVEQLLASIQHLHEGFGGKNILG
JQ765568_DEN/2005/193          ALLNGCRWWLRSTRVNVDGFNEWAMANGYTSVSSVECYSILAAKTGVSVEQLLASIQHLHEGFGGKNILG
JQ765569_DEN/2005/232          ALLNGCRWWLRSTRVNVDGFNEWAMANGYTSVSSVECYSILAAKTGVSVEQLLASIQHLHEGFGGKNILG
JQ765570_DEN/2005/235          ALLNGCRWWLRSTRVNVDGFNEWAMANGYTSVSSVECYSILAAKTGVSVEQLLASIQHLHEGFGGKNILG
JQ765571_DEN/2005/271          ALLNGCRWWLRSTRVNVDGFNEWAMANGYTSVSSVECYSILAAKTGVSVEQLLASIQHLHEGFGGKNILG
JQ765572_DEN/2005/347          ALLNGCRWWLRSTRVNVDGFNEWAMANGYTSVSSVECYSILAAKTGVSVEQLLASIQHLHEGFGGKNILG
JQ765573_DEN/2005/1062         ALLNGCRWWLRSTRVNVDGFNEWAMANGYTSVSSVECYSILAAKTGVSVEQLLASIQHLHEGFGGKNILG
JQ765574_DEN/2005/1862         ALLNGCRWWLRSTRVNVDGFNEWAMANGYTSVSSVECYSILAAKTGVSVEQLLASIQHLHEGFGGKNILG
JQ765575_DEN/2005/1876         ALLNGCRWWLRSTRVNVDGFNEWAMANGYTSVSSVECYSILAAKTGVSVEQLLASIQHLHEGFGGKNILG
JQ765563_DEN/2009/9            ALLNGCRWWLRSTRVNVDGFNEWAMANGYTSVSSVECYSILAAKTGVSVEQLLASIQHLHEGFGGKNILG
JQ765564_DEN/2009/14           ALLNGCRWWLRSTRVNVDGFNEWAMANGYTSVSSVECYSILAAKTGVSVEQLLASIQHLHEGFGGKNILG
JQ765565_DEN/2009/15           ALLNGCRWWLRSTRVNVDGFNEWAMANGYTSVSSVECYSILAAKTGVSVEQLLASIQHLHEGFGGKNILG
JQ765566_DEN/2008/16           ALLNGCRWWLRSTRVNVDGFNEWAMANGYTSVSSVECYSILAAKTGVSVEQLLASIQHLHEGFGGKNILG
JQ765567_DEN/2009/20           ALLNGCRWWLRSTRVNVDGFNEWAMANGYTSVSSVECYSILAAKTGVSVEQLLASIQHLHEGFGGKNILG
```

	290 300
NC_005831_Amsterdam-I	YSSLCDEFTLAEVVKQMYGVNLQ
DQ445911_Amsterdam-057	YSSLCDEFTLAEVVKQMYGVNLQ
DQ445912_Amsterdam-496	YSSLCDEFTLAEVVKQMYGVNLQ
JX104161_CBJ-037	YSSLCDEFTLAEVVKQMYGVNLQ
JX524171_CBJ-123	YSSLCDEFTLAEVVKQMYGVNLQ
JX504050_RPTEC/2004	YSSLCDEFTLAEVVKQMYGVNLQ
KF530110_USA/838-9/1983	YSSLCDEFTLAEVVKQMYGVNLQ
KF530106_USA/8712-17/1987	YSSLCDEFTLAEVVKQMYGVNLQ
KF530114_USA/891-4/1989	YSSLCDEFTLAEVVKQMYGVNLQ
KF530108_USA/891-6/1989	YSSLCDEFTLAEVVKQMYGVNLQ
KF530111_USA/901-24/1990	YSSLCDEFTLAEVVKQMYGVNLQ
KF530109_USA/903-28/1990	YSSLCDEFTLAEVVKQMYGVNLQ
KF530104_USA/904-20/1990	YSSLCDEFTLAEVVKQMYGVNLQ
KF530113_USA/905-25/1990	YSSLCDEFTLAEVVKQMYGVNLQ
KF530107_USA/911-56/1991	YSSLCDEFTLAEVVKQMYGVNLQ
KF530112_USA/0111-25/2001	YSSLCDEFTLAEVVKQMYGVNLQ
KF530105_USA/012-31/2001	YSSLCDEFTLAEVVKQMYGVNLQ
JQ765568_DEN/2005/193	YSSLCDEFTLAEVVKQMYGVNLQ
JQ765569_DEN/2005/232	YSSLCDEFTLAEVVKQMYGVNLQ
JQ765570_DEN/2005/235	YSSLCDEFTLAEVVKQMYGVNLQ
JQ765571_DEN/2005/271	YSSLCDEFTLAEVVKQMYGVNLQ
JQ765572_DEN/2005/347	YSSLCDEFTLAEVVKQMYGVNLQ
JQ765573_DEN/2005/1062	YSSLCDEFTLAEVVKQMYGVNLQ
JQ765574_DEN/2005/1862	YSSLCDEFTLAEVVKQMYGVNLQ
JQ765575_DEN/2005/1876	YSSLCDEFTLAEVVKQMYGVNLQ
JQ765563_DEN/2009/9	YSSLCDEFTLAEVVKQMYGVNLQ
JQ765564_DEN/2009/14	YSSLCDEFTLAEVVKQMYGVNLQ
JQ765565_DEN/2009/15	YSSLCDEFTLAEVVKQMYGVNLQ
JQ765566_DEN/2008/16	YSSLCDEFTLAEVVKQMYGVNLQ
JQ765567_DEN/2009/20	YSSLCDEFTLAEVVKQMYGVNLQ
